# Serotonin receptor type 1B constitutes a therapeutic target for MDS and CMML

**DOI:** 10.1038/s41598-018-32306-4

**Published:** 2018-09-17

**Authors:** Antònia Banús-Mulet, Amaia Etxabe, Josep Maria Cornet-Masana, Miguel Ángel Torrente, María Carmen Lara-Castillo, Laura Palomo, Meritxell Nomdedeu, Marina Díaz-Beyá, Francesc Solé, Benet Nomdedeu, Jordi Esteve, Ruth M. Risueño

**Affiliations:** 1 Josep Carreras Leukaemia Research Institute (IJC), Barcelona, Spain; 2 Faculty of Pharmacy, University of Barcelona, Barcelona, Spain; 3 Institut d’Investigació en Ciències de la Salut Germans Trias i Pujol (IGTP), Badalona, Spain; 4 Faculty of Medicine, University of Barcelona, Barcelona, Spain; 5 Department of Hematology, Hospital Clínic, Barcelona, Spain; 6 Leukos Biotech, Barcelona, Spain; 7 Institut d’Investigacions Biomèdiques August Pi i Sunyer (IDIBAPS), Barcelona, Spain

## Abstract

Myelodysplastic syndromes (MDS) and chronic myelomonocytic leukemia (CMML) are chronic myeloid clonal neoplasms. To date, the only potentially curative therapy for these disorders remains allogeneic hematopoietic progenitor cell transplantation (HCT), although patient eligibility is limited due to high morbimortality associated with this procedure coupled with advanced age of most patients. Dopamine receptors (DRs) and serotonin receptors type 1 (HTR1s) were identified as cancer stem cell therapeutic targets in acute myeloid leukemia. Given their close pathophysiologic relationship, expression of HTR1s and DRs was interrogated in MDS and CMML. Both receptors were differentially expressed in patient samples compared to healthy donors. Treatment with HTR1B antagonists reduced cell viability. HTR1 antagonists showed a synergistic cytotoxic effect with currently approved hypomethylating agents in AML cells. Our results suggest that HTR1B constitutes a novel therapeutic target for MDS and CMML. Due to its druggability, the clinical development of new regimens based on this target is promising.

## Introduction

Myelodysplastic syndromes (MDS) encompass a diverse group of clonal disorders of hematopoietic immature cells characterized by ineffective hematopoiesis. The incidence of MDS in Europe is 1.5/100000 and 5-year survival rate after diagnosis is below 30%^1^. Treatment regimen for MDS mainly depends, beyond patient-related variables, on disease risk stratification, transfusion need and cytogenetic profile^2^. Recently, new treatments such as hypomethylating agents (HMA) and lenalidomide have been approved, with a limited benefit to patients’ outcome^3^.

Chronic myelomonocytic leukemia (CMML) is a myeloid neoplasm with clinical and hematologic features that overlap MDS and myeloproliferative neoplasms (MPN)^4^. Epidemiologically, the median age at diagnosis is approximately 65 years^5,6^ and the disease incidence is approximately 0.3–0.5/100000^1,7,8^. Treatment regimens for CMML include supportive care, HMA, hydroxyurea, AML-type chemotherapy, and, in selected patients, HCT^9^. The overall median survival is inferior to 3 years, with a small improvement in recent years^10–14^. Consequently, there is an unmet need for treatment of these myeloid neoplasms.

The pathogenesis of MDS and CMML is complex and not fully understood. The development of these diseases is a multistep process comprising a severe disturbance within the hematopoietic cell compartment and bone marrow (BM) microenvironment, and the complex interactions between both compartments. Interestingly, approximately one third of MDS and CMML patients develop overt AML during the course of the disease^15^. Mechanisms of disease progression and transformation from a chronic MDS or CMML phase to a more aggressive, usually therapy-resistant AML phase are still poorly understood and the prediction of the transformation is not yet clearly established^16^. On the other hand, the high transformation potential to AML observed in MDS and CMML patients highlights the biological relationship among these hematologic myeloid neoplasms.

Despite the many efforts made by the scientific community to identify new therapeutic targets for MDS and CMML with a clinical significant impact, currently available treatment results in a limited benefit, with HCT arising as the only long-term potential curative therapy^9,17^. Nonetheless, the advanced age and comorbidities of most MDS patients makes HCT an inappropriate option for the majority of patients. Numerous clinical trials testing new agents for MDS and CMML are currently on-going; many of these trials consist of the combination of HMA, the backbone treatment in high-risk MDS, with a novel agent, but, to date, only discrete positive results have been reported^18–22^. Moreover, secondary AML, following MDS and CMML, displays aggressive behavior and poor prognosis. Indeed, the overall survival after transformation is inferior to 6 months^16^. Thus, new therapeutic approaches are desperately required for improved management of myeloid neoplasms.

In the last years, classic neurotransmitter (monoamine) receptors such as dopamine and serotonin receptors have attracted an increasing attention to investigators in oncology^23–29^. Both dopamine receptors (DRs) and serotonin receptors type 1 (HTR1s) are differentially expressed on cancer stem cells, including leukemia stem cells (LSCs), as compared to their normal counterpart; and their inhibition induces differentiation and subsequent cell death of LSCs from primary AML samples in both *ex vivo* and *in vivo* models^23,24^. Interestingly, DRs’ and HTR1s’ signaling disruption also severely affects cell viability of the bulk AML cell population^23,24,30^. Both DRs and HTR1s are G-protein coupled receptors (GPCRs) constituted by seven transmembrane domains. Upon ligand binding, GPCRs suffer a conformation change that result in the activation of G proteins. HTR1 and DRD2/3/4 are Gα_i_-coupled GPCRs; thus, activation of the receptor inhibits the production of cAMP by adenylate cyclase. Activation of DRD1/5 may either transduce activation signaling through Gα_o_ or Gα_s_. Thus, whereas Gα_s_ stimulates cAMP production, Gα_o_ inhibits its production; the activation of either Gα protein depends on the cellular context.

Here, we demonstrate that HTR1s and DRD3/5 are differentially expressed on MDS and CMML cells, as compared to their normal hematopoietic counterparts. HTR1, especially HTR1B, behaves as a promising therapeutic target for both MDS and CMML, similarly to AML. Inhibition of HTR1B reduces cell viability and displays an interesting synergistic anti-neoplastic effect when combined with currently approved HMAs (azaciditine or decitabine), at least in AML cells. On the other hand, DR inhibition results in a reduction in cell viability of AML and CMML cells but not MDS samples. Interestingly, the anti-leukemic effect observed with HTR1 antagonists is enhanced in the presence of DR antagonists, suggesting the existence of a cross-talk between both types of receptors, at least in myeloid neoplasms. Our results support a further clinical development of novel treatment strategies based on HTR1B antagonists in combination with DRs’ antagonists for myeloid neoplasms.

## Results

### HTR1s and DRs are differentially expressed in MDS and CMML patient cells, similarly to AML

**Figure 1:**
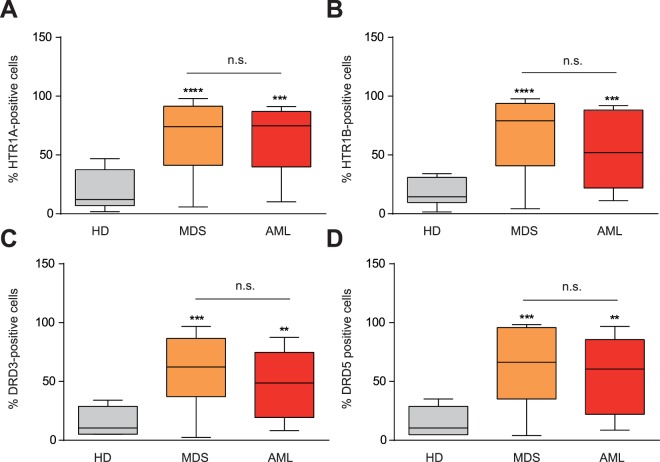
HTR1A/B and DRD3/5 are expressed in AML and MDS. (**A**) HTR1A (HD n = 6; MDS n = 54; AML n = 14), (**B**) HTR1B (HD n = 6; MDS n = 55; AML n = 14), (**C**) DRD3 (HD n = 4; MDS n = 47; AML n = 14), and (**D**) DRD5 (HD n = 4; MDS n = 47; AML n = 14) surface expression measured by flow cytometry in blood samples from healthy donors (HD), MDS samples and AML samples. Frequency of positive cells for each marker is graphed as a box-and-whisker (turkey) plot, the statistical median is indicated as a horizontal line, error bars correspond to SEM and boxes indicated the lowest and upper quartile. **p < 0.01; ***p < 0.001; ****p < 0.0001.

Due to the key role that HTR1s and DRs play in AML^23,24^ and the close physiopathological relationship between AML and MDS, the expression of HTR1s and DRs was screened by flow cytometry in BM samples from MDS patients. Similar to AML, MDS patient samples highly expressed HTR1A (Fig. 1A) and HTR1B (Fig. 1B) on the cell surface. The expression of HTR1A and HTR1B on MDS BM cells was 3.4- and 3.7-fold higher compared to healthy donor samples (HD), respectively. Interestingly, expression levels of HTR1A and HTR1B were comparable in MDS and AML patients.

Similarly, DRD3 and DRD5 were highly expressed in MDS and AML patient samples as compared to HD blood cells (Fig. 1C,D). The expression of both DRs was equivalent, displaying a 3.5-fold increase with respect to HD cells. Although all samples expressed DRs on the surface, only DRs type 3 (DRD3) and 5 (DRD5) were significantly higher expressed than HD blood cells (Figure S1). Moreover, DRD3 and DRD5 expression have been comprehensively studied in the context of AML^23^. Consequently, DRD3 and DRD5 were chosen for further validation as biomarkers for MDS cells.

**Figure 2:**
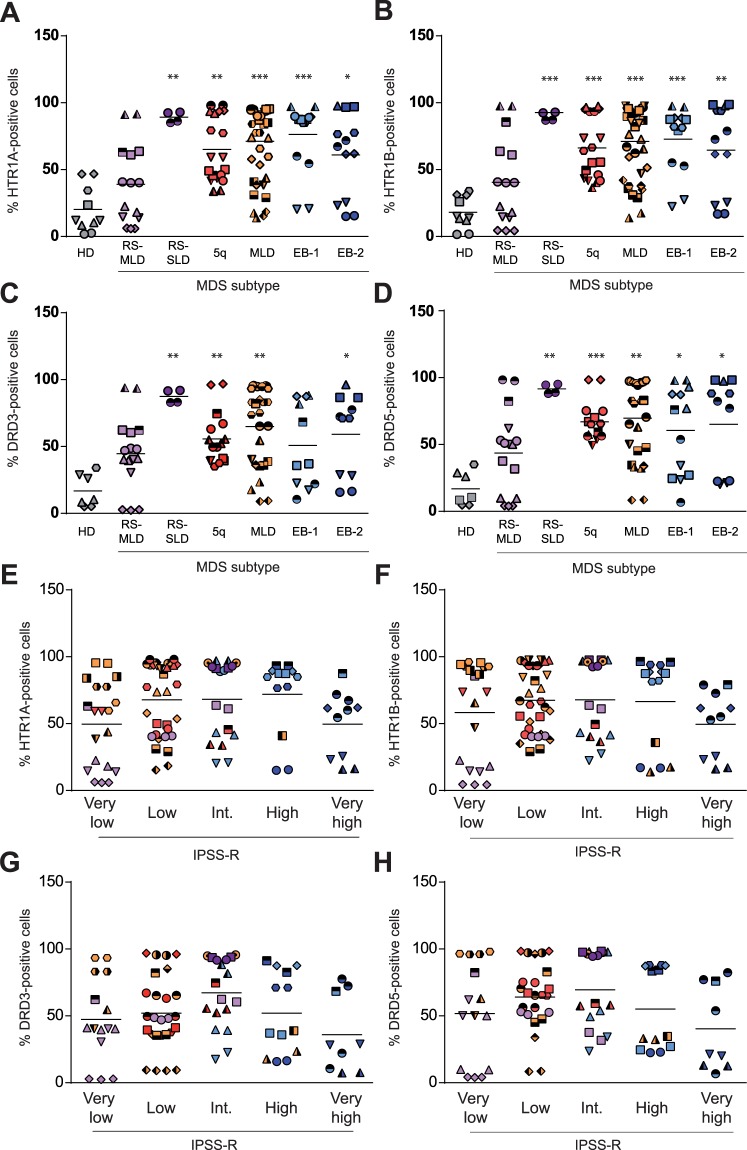
All MDS subtypes differentially express DRD3/5 and HTR1A/B, except MDS-RS-MLD. MDS patient samples tested for (**A**) HTR1A, (**B**) HTR1B, (**C**) DRD3 and (**D**) DRD5 surface expression by flow cytometry. Each subtype of MDS is represented (Healthy donor (HD), grey; RS-MLD, violet; RS-SLD, purple; 5q, red; MLD, orange; EB-1, light blue; EB-2, dark blue). MDS patient samples were classified according to IPSS-R (Very low, low, intermediate, high, very high) and the surface expression of (**E**) HTR1A, (**F**) HTR1B, (**G**) DRD3 and (**H**) DRD5 is represented. Frequency of positive cells is graphed. Each symbol type corresponds to a patient sample, and each symbol corresponds to an experimental point. Grand mean values are indicated with horizontal lines. *p < 0.05; **p < 0.01; ***p < 0.001; ****p < 0.0001.

MDS is driven by a complex combination of genetic and epigenetic changes that result in a wide heterogeneity in both clinical phenotype and disease outcome. According to the World Health Organization (WHO) classification^4^, MDS is a clonal disease characterized by morphological dysplasia, ineffective hematopoiesis leading to cytopenias and risk of transformation to AML^4^. MDS is subclassified into clinically relevant groups mainly based on morphological and cytogenetic criteria^4^. Thus, we next interrogated the expression of HTR1A/B and DRD3/D5 within the most frequent MDS subtypes (Figure S2). As shown in Fig. 2A–D, the expression of HTR1A, HTR1B, DRD3 and DRD5 was significantly higher in all MDS subgroups as compared to HD samples, with the exception of MDS-RS (MDS with ring sideroblasts) with multilineage dysplasia (MDS-RS-MLD), which showed only an increase in DRD3 expression. Indeed, the frequency of positive cells for HTR1s and DRs was above 50% in MDS-RS with single lineage dysplasia (MDS-RS-SLD); MDS with isolated del(5q) (MDS-5q); MDS with multilineage dysplasia (MDS-MLD); and MDS with excess blasts type 1 (MDS-EB-1) and type 2 (MDS-EB-2) (Fig. 2A–D) (Table 1). Notably, MDS-RS-MLD samples expressed HTR1s and DRs similarly to HD samples. Due to the cell subpopulation heterogeneity within MDS samples, the expression of HTR1A/B and DRD3/5 was also interrogated in the CD34-positive cell population, which corresponds to the most primitive fraction. Expression levels of each receptor were similar in the bulk population and CD34^+^ fraction (Figure S3). Indeed, CD34-positive HD cells lacked the expression of HTR1A/B (Figure S3) and DRD3/5^23^.

**Table 1:** DR and HTR expression in MDS samples.

	Mean	Lower-Upper 95% CI	SEM	pvalue
**RS-MLD**				
HTR-1A	48,17	20,62–75,71	11,65	0,0954
HTR-1B	45,48	12,91–78,05	13,31	0,1224
DRD3	49,94	23,41–76,46	10,84	0,0772
DRD5	50,03	19,71–80,36	12,39	0,1126
**RS-SLD**				
HTR-1A	89,15	44,68–133,6	3,5	0,0017
HTR-1B	89,83	53,93–125,7	2,825	0,0003
DRD3	87,4	32,13–142,7	4,35	0,0032
DRD5	91,55	51,53–131,6	3,15	0,0027
**5Q-**				
HTR-1A	63,77	46,22−81,31	7,754	0,0023
HTR-1B	65,4	48,86–81,93	7,309	0,0006
DRD3	55,97	39,64–74,29	7,327	0,0084
DRD5	66,51	54,09–78,94	5,255	0,0004
**MLD**				
HTR-1A	69,24	57,22–81,26	5,794	0,0004
HTR-1B	68,82	56,94–80,69	5,712	0,0002
DRD3	63,96	44,22–78,69	6,951	0,0072
DRD5	68,33	53,49–83,18	7,001	0,0041
**EB-1**				
HTR-1A	76,64	55,31–97,97	9,02	0,0006
HTR-1B	73,25	49,89–96,61	9,546	0,0007
DRD3	52,25	18,57–85,93	13,1	0,0904
DRD5	61,58	32,17–90,98	12,02	0,0348
**EB-2**				
HTR-1A	63,25	33,13–93,36	12,31	0,164
HTR-1B	66,99	35,18–91,79	13	0,0092
DRD3	62,26	28,23–96,29	13,24	0,038
DRD5	67,74	29,59–105,9	14,84	0,0367
**Healthy donors**				
HTR-1A	21,38	3,708–39,04	6,873	
HTR-1B	20,03	6,961–33,1	5,085	
DRD3	18,98	−3,183–41,15	6,965	
DRD5	19,14	−3,803–42,08	7,209	

Frequency of positive cells (mean value) is indicated. CI: confidence interval. SEM: Standard error of the mean.

The natural history of MDS varies considerably among individuals, which correlates with the mosaic of subtypes of MDS^3^. In order to better discriminate prognostic risk for assessing clinical outcomes in MDS, IPSS-R was developed based on five disease factors (percentage of blasts, cytogenetics, hemoglobin concentration, absolute neutrophil and platelet count) that categorized MDS patients into five risk categories: very low, low, intermediate, high, and very high^31^. Therefore, we further analyzed if the expression of HTR1A, HTR1B, DRD3 and DRD5 correlated with the diverse IPSS-R prognostic categories. As shown in Fig. 2E–H, the expression of HTR1A, HTR1B, DRD3 and DRD5 was independent of prognostic-risk, since no significant correlation was observed between the surface expression of these receptors and any of the IPSS-R prognostic groups.

### Inhibition of HTR1B in MDS cells induces cytotoxicity

**Figure 3:**
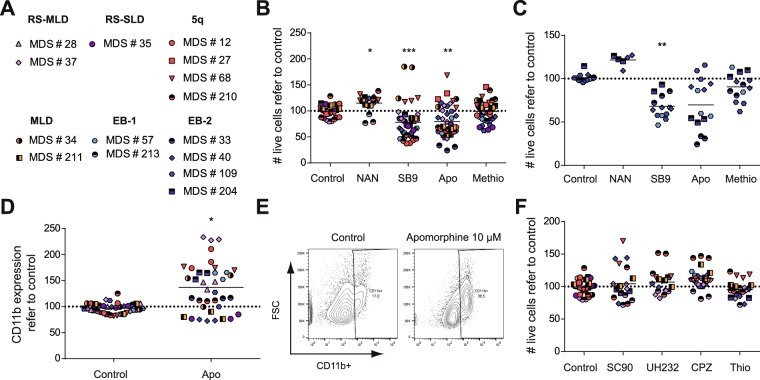
Treatment with HTR1 antagonists reduces MDS cell viability. (**A**) MDS patient samples used in cytotoxic experiments classified by subtypes. (**B**) MDS patient samples or (**C**) specifically MDS-EB-1/2 samples were treated with 10 µM of subtype specific-HTR antagonists (apomorphine –apo-, HTR1/2; methiothepin –methio-, HTR1/2; NAN190 –NAN-, HTR1A; SB-224289 –SB9–, HTR1B) for 72 h and cell viability was measured by 7-AAD exclusion by flow cytometry. Normalized live cell counts against vehicle-treated samples are represented. (**D**) MDS patient samples were treated with 10 µM apomorphine for 72 h and the expression of the granulocytic associated-differentiation surface marker CD11b was measured by flow cytometry. Normalized frequency of positive cells refer to vehicle control-treated samples is represented. (**E**) CD11b surface expression upon treatment with 10 μM apomorphine is shown. (**F**) MDS patient samples were treated with 10 µM of subtype specific-DR antagonists (SCH-23390 –SC90–, DRD1/5; UH-232 –H232–; DRD2/3; Chlorpromazine –CPZ–; pan-DR, Thioridazine –Thio–, pan-DR) for 72 h and cell viability was measured by 7-AAD exclusion by flow cytometry. Each symbol type corresponds to a patient sample, and each symbol corresponds to an experimental point. Grand mean values are indicated with horizontal lines. *p < 0.05; **p < 0.01; ***p < 0.001; ****p < 0.0001.

HTR1s have been recently described as therapeutic targets in AML^24^. Indeed, inhibition of HTR1A and HTR1B leads to a reduction in cell viability coupled with the induction of the differentiation program of AML cells both *ex vivo* and *in vivo*^24^. Since MDS samples differentially expressed HTR1A and HTR1B similarly to AML (Figs 1 and 2), their sensitivity to HTR1A/B antagonists was evaluated *ex vivo*. MDS samples corresponding to MDS-RS-MLD (violet), MDS-RS-SLD (purple), MDS-5Q (red), MDS-MLD (orange) and MDS-EB1/2 (blue) were treated for 72 h with apomorphine (a promiscuous dual HTR1/2 antagonist and a DR partial agonist), methiothepin (a broad-spectrum HTR antagonist), SB-224289 (a highly selective HTR1B antagonist), and NAN-190 (a highly selective HTR1A antagonist), and subsequent cell viability and the differentiation stage were measured (Fig. 3B). A wide range of cell response to the different agents assayed was observed, in accordance with the intrinsic heterogeneity within MDS disease behavior among patients. An overall statistically significant but clinically irrelevant reduction in cell viability was observed upon apomorphine and SB-224289 treatment (Fig. 3B); cellular responses to methiothepin were heterogeneous, probably due to a lower affinity. On the contrary, NAN-190, the selective HTR1A antagonist, spared MDS samples in terms of cell survival (Fig. 3B). When the analysis was focused on MDS-EB-1/2 samples (blue), a higher sensitivity to HTR1 antagonists was observed (Fig. 3C) (cell viability mean for apomorphine: 80.53 ± 7.243 in MDS vs. 68.48 ± 11.34 in MDS-EB-1/2; methiothepin: 99.61 ± 4.799 in MDS vs. 89.88 ± 5.394 in MDS-EB-1/2; SB-224289: 78.26 ± 8.749 in MDS vs. 66.36 ± 6.11 in MDS-EB-1/2). Both broad HTR1/2 antagonists, apomorphine and methiothepin, triggered cell death in MDS-EB1/2 samples, whereas the difference observed in methiothepin was not statistically significant. Again, the highly specific HTR1B antagonist SB-224289 induced the greatest reduction in cell viability in MDS-EB samples. Similar to AML^24^, treatment with apomorphine and SB-224289 induced the expression of the differentiation-associated marker CD11b (Figs 3D–E and S4A,B). Interestingly, those MDS subgroups that displayed the highest resistance to HTR1 antagonists (MDS-5Q and MDS-RS-MLD) showed the highest differentiation induction upon treatment (CD11b expression in MDS-5Q: 143.70 ± 20.22; MDS-RS-SLD: 79.35 ± 2.774; MDS-MLD: 138.10 ± 19.31; MDS-RS-MLD: 186.40 ± 43.79; MDS-EB-1/2: 116.10 ± 17.85). Therefore, inhibition of HTR1 affected cell viability and differentiation status in MDS cells.

Similarly to HTR1 antagonism^24^, DR antagonism in AML produces a reduction in cell viability coupled with the initiation of the differentiation program^23^. We therefore interrogated the cellular effect of the treatment with DR antagonists such as SCH-23390 (SC90) (DRD1 and DRD5 antagonists), UH-232 (DRD2 and DRD3 antagonist), chlorpromazine (CPZ) (pan-DR antagonist), and thioridazine (Thio) (pan-DR antagonist) in MDS. Surprisingly, and in contrast to AML^23^, none of the DR antagonists was cytotoxic for MDS samples (Fig. 3F), even though DRs were overexpressed on these patient cells. Only thioridazine at a 50 μM concentration produced a therapeutic significant induction of cell death (Figure S4C); however, at this concentration, thioridazine has been proven toxic to normal cells^23^. In contrast to HTR1B antagonists, DR antagonists showed neither cytotoxicity nor induction of differentiation in any MDS subgroup (Figure S4B,C).

**Figure 4:**
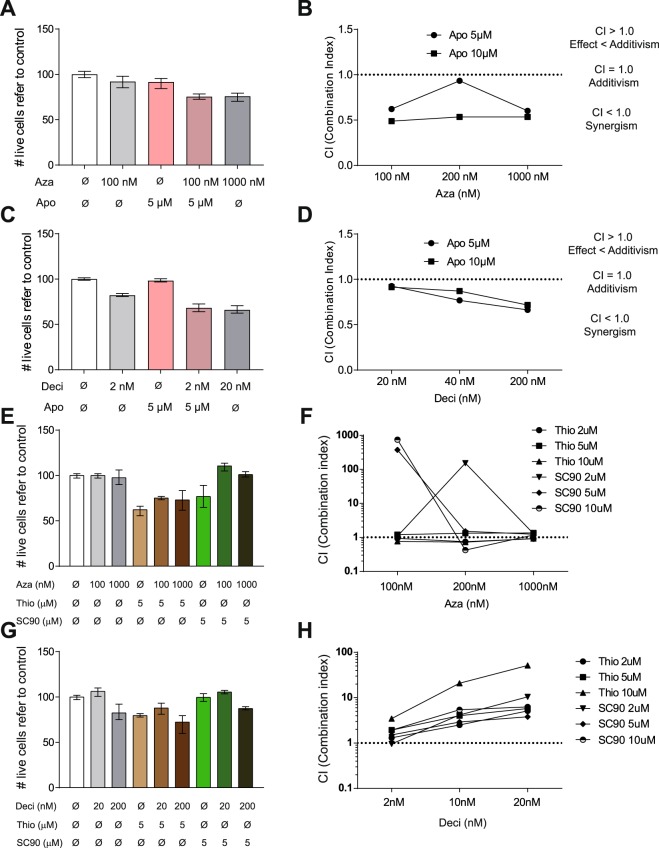
HTR and DR antagonist shown synergism with HMAs. MonoMac-1 AML cells were treated for 72 h with apomorphine –apo– (5 and 10 µM) and azaciditine –aza– (100, 200 and 1000 nM). (**A**) Cell viability was measured by 7-AAD exclusion by flow cytometry. (**B**) Synergism between drugs was evaluated based on the combination index method (CI). MonoMac-1 AML cells were treated for 72 h with apomorphine –apo– (5 and 10 µM) and decitabine –deci– (20, 40 and 200 nM). (**C**) Cell viability was measured by 7-AAD exclusion by flow cytometry. (**D**) Synergism between drugs was evaluated based on the combination index method (CI). (**E**) MonoMac-1 AML cells were treated for 72 h with thioridazine –thio– or SCH-23390 –SC90– (2, 5 and 10 µM) and azaciditine –aza– (100, 200 and 1000 nM). Cell viability was measured by 7-AAD exclusion by flow cytometry. (**F**) Synergism between drugs was evaluated based on the combination index method (CI). MonoMac-1 AML cells were treated for 72 h with thioridazine –thio– or SCH-23390 –SC90– (2, 5 and 10 µM) and decitabine –deci– (20, 40 and 200 nM). (**G**) Cell viability was measured by 7-AAD exclusion by flow cytometry. (**H**) Synergism between drugs was evaluated based on the combination index method (CI). Bars represent mean values of triplicates. Error bars represent range.

Recently, HMAs such as azaciditine and decitabine have shown a clinical benefit for high-risk MDS patients, becoming a major advance in the treatment of these patients. However, 50% of MDS patients are non-responders and the majority of responders relapse within 2 years^32^. As both agents act on proliferative cells (S-phase) and treatment with HTR and DR antagonists induces cell cycle entry^23,24^, a potential synergistic cytotoxic effect of the combination of HTR1 antagonists and HMAs was investigated. To date, only 2 MDS cell lines have been generated: MDS92^33^ and its derivative MDS-L^34,35^. MDS-L successfully reproduces the disease in xenograft models^34^. However, the expression of DRs and HTR1s was absent in these cell lines, in contrast to primary MDS patient samples (Figure S5). Therefore, the pharmacologic interaction between HTRs antagonists and HMAs was analyzed in AML cells that express DRs and HTRs similarly to MDS (Fig. 1), which express DRs and HTR1s similarly to MDS primary samples. The presence of apomorphine synergistically interacted with azaciditine inducing cytotoxicity (Fig. 4A,B). Indeed, 100 nM azaciditine in the presence of 5 μM apomorphine induced equivalent cell death level as 1 μM azaciditine (Fig. 4A). Similarly, decitabine treatment also showed synergistic anti-leukemic effect in combination with apomorphine (Fig. 4C,D). Moreover, synergism between drugs could be demonstrated based on both combination index (CI)^36^ (Fig. 4B,D) and Excess Over Bliss additivism (EOBA)^37^ (Figure S6A,B). Indeed, equivalent results were obtained with methiothepin in combination with HMAs (Figure S6C,D). Next, interaction between different DR antagonists with the HMAs was evaluated similarly to HTR1 antagonists. In concordance to the cytotoxicity data observed with the use of DR antagonists alone, the anti-neoplastic effect of azaciditine (Fig. 4E,F) and decitabine (Fig. 4G,H) was not potentiated in the presence of the DR antagonists thioridazine (Thio) and SCH-224289. Taken together, HTR1 constitutes a potential therapeutic target in combination with currently approved HMAs in MDS.

### CMML cells are sensitive to both HTR1B and DRs’ antagonists

**Figure 5:**
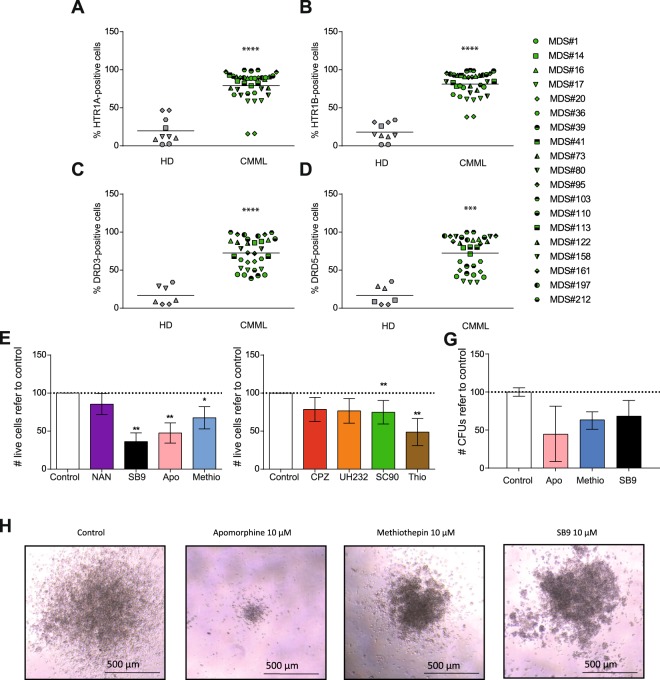
DRs and HTR1s are differentially express on CMML samples. CMML patient samples and healthy blood cells (HD) were tested for (**A**) HTR1A, (**B**) HTR1B, (**C**) DRD3 and (**D**) DRD5 surface expression by flow cytometry. Frequency of positive cells is represented. Each symbol type corresponds to a CMML patient samples, each symbol corresponds to an experimental point. Grand mean values are shown as a horizontal line. CMML patient samples were treated for 72 h with 10 µM of (**E**) subtype specific-HTR antagonists (apomorphine –apo–, HTR1/2; methiothepin –methio–, HTR1/2; NAN190 –NAN–, HTR1A; SB-224289 –SB9–, HTR1B) and 10 μM of (**F**) subtype specific-DR antagonists (SCH-23390 –SC90-, DRD1/5; UH-232 –H232-, DRD2/3; Chlorpromazine –CPZ-, pan-DR; Thioridazine –Thio-, pan-DR) and cell viability was measured by 7-AAD exclusion by flow cytometry. Data is normalized against vehicle-treated control sample. CMML n = 4 in triplicates. (**G**) CMML patient sample were plated in methylcellulose and the number of CFUs refer to control is represented. Normalized data refer to control are represented as mean ± range. CMML n = 2 in duplicates. *p < 0.05; **p < 0.01; ***p < 0.001; ****p < 0.0001.

CMML is a clonal hematopoietic stem cell disorder, characterized by overlapping features between MDS and myeloproliferative neoplasms, with an inherent tendency to transform to AML^38^. Based on these characteristics, the expression of HTR1 and DR was also analyzed in CMML patient samples. Similar to MDS, CMML samples differentially expressed HTR1A (Fig. 5A), HTR1B (Fig. 5B), DRD3 (Fig. 5C), and DRD5 subtypes (Fig. 5D). Next, sensitivity to HTR1 antagonists’ treatment was assayed in similar conditions as described for MDS samples. In concordance with the expression profile, apomorphine, methiothepin, and SB-224289 reduced cell viability at 10 μM (Fig. 5E). In contrast to MDS, CMML samples were also sensitive to treatment with DR antagonists (such as CPZ, UH-232, SCH-23390 and Thio) (Fig. 5F). Interestingly, the inhibition of HTR1B resulted in the reduction of the clonogenic capacity of CMML samples (Fig. 5G–H). These results suggest that HTR1B and DRs may also act as a therapeutic target for CMML analogously to AML.

### Simultaneous inhibition of HTR1 and DR enhances the cytotoxic effect

**Figure 6:**
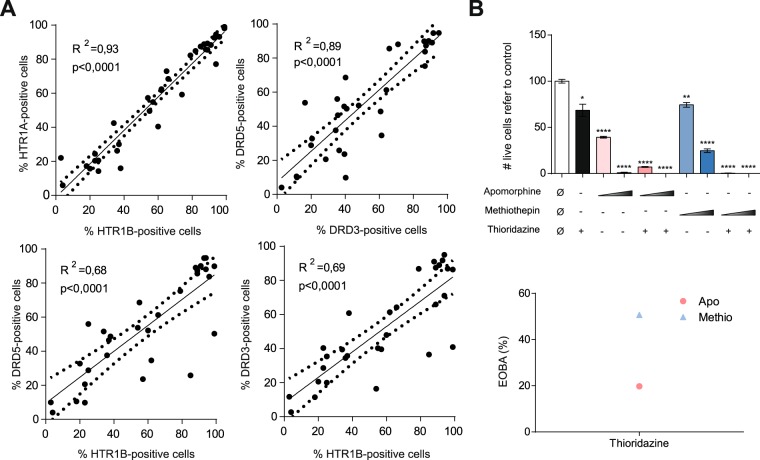
DR3/5 and HTR1A/B are co-expressed in MDS and CMML cells. (**A**) Frequency of HTR1A- vs. HTR1B- (upper left), DRD3 vs. DRD5 (upper right), HTR1A vs. DRD5 (lower left) and HTR1B vs. DRD5 (lower right)-positive cells are represented. The coefficient of determination measured as R^2^ is specified. The regression line is shown as a solid line; the confidence interval is represented as a dotted line. (**B**) HL-60 AML cell line was treated for 72 h with apomorphine (5 µM and 10 µM) and methiothepin (5 µM and 10 µM), in combination with thioridazine at 10 µM. Cell viability was measured by 7-AAD exclusion by flow cytometry (left panel). The synergistic effect in combination treatment measured was evaluated based on EOBA (Excess Over Bliss Additivism) (right panel). *p < 0.05; **p < 0.01; ***p < 0.001; ****p < 0.0001.

Oligomerization is a general characteristic of GPCRs, and homo- and heterodimerizations are found in DRs and HTR1s^39^. Actually, DRs/HTR1s complexes have been described^40^. The HTR and DR expression patterns observed in MDS samples were comparable (Figs 1 and 2). Indeed, HTR1A, HTR1B, DRD3 and DRD5 were expressed in each sample at the same level in both MDS and CMML (Fig. 6A). The cytotoxic effect observed upon treatment with HTR1 antagonists and DR antagonists was potentiated between 20–50% in co-treatment (Fig. 6B) in AML cell lines; thus, HTR1 and DR antagonists presented a synergistic anti-leukemic effect. Therefore, HTR1s and DRs seem to cooperate in the survival and/or proliferation of leukemic cells.

## Discussion

The overexpression of HTR1 and DRs in MDS and CMML, described in this study, was also recently described in AML patients^23,24^. Regardless of disease subtype, these biomarkers arose as potential therapeutic targets in both disorders. Indeed, HTR1B antagonists induced cytotoxicity and differentiation of MDS and CMML cells; whereas DR antagonists showed an antiproliferative effect in CMML, in contrast to MDS cells. Interestingly, HTR1 antagonists displayed a synergistic effect with HMAs, which currently constitute the essential treatment for high-risk MDS patients; although due to technically difficulties, the synergist effect was studied in AML cells. The *in vitro* activity observed with these agents warrant their translation to clinical trials, given the incurability of these diseases with currently approved therapies and, therefore, the urgent need of new therapeutic strategies for this challenging group of patients.

HTR1B and DR3/5 receptors were consistently upregulated in a wide series of MDS patients diagnosed with different MDS subtypes, similar to previous description in AML patients^23,24^, and this finding was also observed among CMML specimens. Moreover, given the profound effect of HTR1B inhibition on cell viability and differentiation, the distinctive higher expression of these receptors in comparison to normal hematopoietic cells supports the key role of HTR1B signalling in the pathogenesis of these myeloid diseases. The lack of clinical correlation with HTR1B and/or DR expression across patient samples imply that these signaling pathways might not be involved in mechanisms governing disease progression and/or AML transformation.

The antineoplastic activity observed with different types of HTR antagonists identified HTR1B as the key target for further exploring for clinical purposes, either using a wide-spectrum agent as apomorphine or a more selective HTR1B antagonist. Of note, reduction of cell viability parallel induction of differentiation, supporting the rationale of differentiating therapy in MDS patients^41^.

Although AML, MDS and CMML are myeloid neoplasms that share common features such as an impaired terminal differentiation of mature cells, the intimate pathophysiology responsible and disease phenotype is remarkably diverse. AML is characterized by a marked block in differentiation coupled with an increased proliferation kinetics of leukemic progenitors that results in the accumulation of blasts in BM and peripheral blood. On the contrary, in MDS, the lack of terminal normal mature cells is mainly attributed to a failure of differentiation. CMML is a highly diverse entity, which combines dysplastic features resembling MDS with proliferative traits, especially evident in some patients with hyperleukocytosis. These discrepancies are probably due to differences in cell cycle status, cell survival and cell proliferation rate^42^. Accordingly, the degree of cell viability reduction of AML and CMML cells upon treatment with HTR1 and DR antagonists was similar. In contrast, MDS cells were more sensitive to HTR1 antagonists, as compared to DR antagonists, even though the expression level of these biomarkers was equivalent across samples. These results suggest that DRs may be implicated mainly in the block of differentiation observed in CMML and AML; whereas HTR1 may play a critical role in survival of myeloid-transformed blasts. Alternately, formation of HTR1/DR oligomers may constitute a fine-tuning signaling regulation that mediates leukemogenesis.

Due to the characteristic intraclonal and interpatient heterogeneity of myeloid neoplasms, monotherapy regimens are unlikely to be capable of a durable, clinically meaningful disease control. Therefore, combination therapies that may target the different neoplastic subpopulation are highly desirable. DR and HTR1 antagonists not only act synergically as anti-leukemic agents, but also positively interact with currently approved HMAs (azaciditine and decitabine), the current cornerstone in MDS treatment, fully justify the design of clinical trials exploring combination of these diverse drugs. Interestingly, the concentrations used are within the safety range, as previously published^23,24^. Moreover, dopamine and serotonin blockage are widely used among patients with Parkinson’s disease without remarkable side effects^43^, and this clinical experience could facilitate their repositioning as new agents for myeloid neoplasms^44^. Intriguingly, patients with Parkinson’s disease have been reported to present a lower incidence of hematological cancers^45,46^; although this epidemiological observation and putative casualty with generalized, long-standing use of DR and HTR antagonists should be prospectively addressed.

In conclusion, the differential expression of HTR1B and DRs in MDS and CMML cells, together with the observed effect on cell viability and differentiation induction upon HTR1B and DR inhibition in patient specimens identify these monoamine receptors as potential therapeutic targets in myeloid neoplasms. Moreover, the well proven clinical safety of DR and HTR antagonists and the synergic potential in combination with HMAs justify their rapid translation to clinical experimentation.

## Methods

### Primary samples

Primary MDS and CMML samples were obtained from patients diagnosed with MDS and CMML at Hospital Clínic of Barcelona (Spain) and Hospital Germans Trias i Pujol (Badalona, Spain). Alternatively, MDS and CMML samples were obtained from the Sample collection located at the Hematology Laboratory of IJC (ref#C0000886). MDS and CMML diagnosis and classification was based on standard WHO criteria^4,47^. Main MDS/CMML patient’s characteristics are summarized in Tables 2 and S1. Samples were obtained from bone marrow and mononuclear cells (MNCs) were isolated by Ficoll density gradient centrifugation (GE). All patients provided written informed consent in accordance with the Declaration of Helsinki, and the study was approved by the Ethics Committee of Hospital Clínic of Barcelona and Hospital Germans Trias i Pujol; thus, all methods were performed in accordance with relevant guidelines and regulations. Healthy donor samples were obtained from the Banc de Sang i Teixits of Barcelona (Spain).

**Table 2:** Patients’ information.

Sample code	Age	Gender	WHO 2016	Karyotype	IPSS-R	% Blasts BM
#1	76	M	CMML-1	46,XY[20]	N/A	1
#5	83	M	MDS-EB-1	47,XY, +8[3]/46,XY[28]	High	7
#6	46	F	RS-MLD	46,XX[20]	Low	2
#9	70	F	RS-MLD	46,XX[20]	Int	4
#10	74	M	MDS-EB-2	45,X, −Y,del(1)(p13p32),der(11)t(Y;11)(q11;q13)[8]/46,XY[12]	Very high	12
#12	73	F	MDS with isolated del(5q)	46,XX,del(5)(q22q33)[15]/46,XX[5]	Low	2
#14	72	M	CMML-0	46,XY,del(5)(q31q33)[6]	N/A	0
#15	51	M	MLD	N/A (normal FISH 5p15.2, 5q31, 7q31 & 20q12)	N/A	2
#16	59	F	CMML-0	46,XX[20]	N/A	2
#17	79	M	CMML-2	46,XX[20]	N/A	13
#20	52	M	CMML-1	46,XY[20]	N/A	4
#23	71	F	MDS with isolated del(5q)	46 XX, −11, +mar [20]	Int	3
#24	29	F	MDS isolated del(5q)	46,XX,del(5)(q12q32)[11]/46,XX[9]	N/A	N/A
#26	81	F	MLD	46,XX[10]	Low	0
#27	59	F	MDS with isolated del(5q)	46,XX,del(5)(q13q33)[4]/46,XX[15]	Low	2
#28	65	M	RS-MLD	46,XY[20]	Very low	2
#29	86	F	MLD	47,XX, +8[10]/48,idem, +mar[2]/46,XX[7]	High	3
#30	65	M	RS-MLD	46,XY[20]	Very low	2
#31	71	M	MDS with isolated del(5q)	46,XY,del(5)(q14q34)[8]/46,XY[22]	Low	7
#32	78	M	MDS with isolated del(5q)	46,XY,del(5)(q13q33)[12]/46,XY[8]	Very low	2
#33	77	M	MDS-EBS2	Complex	Very high	>5%
#34	67	M	MDS-MLD	46,XY[20]	Low	4
#35	78	F	MDS-RS	46,XX[20]	Very low	0
#36	60	M	CMML-1	N/A	N/A	0
#37	60	F	RS-MLD	46,XX[20]	Very low	1
#38	91	M	MDS-EB-1	45,X, −Y[14]/46,XY[6]	Int	7
#39	70	F	CMML-1	46,XX[20]	N/A	1
#40	67	M	MDS-EB-2	43,XY, −5,der(13;14)(q10;q10),add(15)(p10),add(16)(q24), −17,add(17)(p13), −18, +mar[cp14]	Very high	15
#41	71	M	CMML-0	46,XX[20]	N/A	0
#43	83	M	MDS-MLD	46,XY[20]	Very low	1
#49	67	F	MDS-EB1	26/08/2015: 46,XX,del(5)(q11q31)[15]	Low	8
#50	75	M	MDS-MLD	47,XY, +21[5]/46,XY[15	Low	2,5
#52	78	F	MDS-MLD	46,XX,del(5)(q22q35),del(11)(q13.1q23.3)[17]/46,XX[3]	Low	3,5
#53	56	M	MDS-MLD	46,XY[20]	Low	2
#57	79	M	MDS-EB-1	46,XY[21]	Int	5
#60	80	F	MDS with isolated del(5q)	46,XX,del(5)(q13)[11]/46,XX[9]	Low	4
#68	94	F	MDS with isolated del(5q)	46,XX,del(5)(q13q33)[9]/46,XX[24]	Very low	1
#73	76	M	CMML-0	46,XY[20]	Very low	1,5
#74	80	M	MDS-MLD	46,XY[20]	Low	0
#76	83	F	MDS-MLD	del(5q)	Low	<5
#78	73	F	MDS-MLD	46,XX,add(9)(p24),16qh+c[7]/46,XX,16qh+c[7]	Low	<2
#80	67	F	CMML-2	46,XX[20]	Int	13
#93	79	F	RS-SLD with thrombocytosis	N/A	Low	1
#95	66	M	CMML-0	46,XY,t(4;16)(q21;q24)[3]/47,sl, +8[17]	N/A	0
#98	63	F	MDS with isolated del(5q)	del(5q) add(7)	Low	1
#103	70	M	CMML-1	46,XY[20]	N/A	N/A
#110	80	M	CMML-1	46,XY[20]	N/A	N/A
#113	56	M	CMML-1	47,XY, +8[14]/46,XY[3]	N/A	N/A
#119	53	F	MDS-EB1	46,XX,del(5)(q14q33)[7]/46,XX [13]	Int	7,8
#122	N/A	F	CMML-1	47,XX, +21[13]	N/A	N/A
#131	68	F	MDS-MLD	46,XX[20]	Very low	0
#132	72	M	MDS-MLD	46,XY[20]	Very low	<2
#134	45	M	MDS-MLD	46,XY[20]	Low	1
#138	68	F	MDS-MLD	46,XX,del(5)(q13q33),del(11)(q13q23)	Low	1
#140	69	F	MDS-RS-SLD	(−7)	N/A	N/A
#143	69	M	MDS/MPN (MDS/MPN-RS-T)	46,XY[13]	Low	<2
#145	83	M	MLD	45,X, −Y[18]/46,XY[2]	Low	3
#149	64	F	MDS with isolated del(5q)	46,XX del(5)(q15q33)[20]	Low	1,6
#151	53	F	MDS-EB2	N/A	High	12
#154	79	F	MDS-EB-1	46,XX[20]	Int	6
#161	69	M	CMML-1	46,XY[20]	N/A	N/A
#162	54	M	MDS-EB2	46,XY,t(16;17)(q24;q22)	Very high	12
#166	73	M	MDS-MLD	46,XY[2]	Low	2
#170	58	M	MDS-SLD	46,XY,inv(2)(p23q13),del(5)(q13q32)	Low	1
#176	70	F	MDS-MLD	46,XX[20]	Very low	1
#181	73	F	MDS-MLD	46,XX,add(9)(p24),16qh+c[7]/46,XX,16qh+c[7]	Low	<2
#186	62	M	MDS-MLD	45,X,(−Y)[10]/46,XY[1]	Very low	1
#187	84	F	MDS-MLD	46,XX[14]	Very low	2,1
#188	80	M	MDS-MLD	46,XY[20]	Low	0
#195	72	M	MDS-MLD	46,XY[20]	Very Low	<2
#197	N/A	M	CMML	N/A	N/A	N/A
#210	59	F	MDS with isolated del(5q)	46,XX,del(5)(q22q31–32)[5]/46,XX[15]	Low	2
#211	75	F	MLD	46,XX, −5, −14, +mar1, +mar2[16]/45,XX,del(5)(q13q33), −6[2]/46,XX[8]	High	1
#212	66	M	CMML-0	46,XY[20]	N/A	4
#213	83	F	MDS-EB-1	del(7)(q22q31)	Very high	5
#214	30	F	MLD	46,XX[20]	Vey low	2

M, male; F, female. Int, intermediate. N/A, not available.

### AML cell lines and cell cultures

AML cell lines HL-60 (ACC-3), and MonoMac-1 (ACC-252) were obtained from DSMZ. MDS-L^34,35^ was kindly provided by Dr. Starczynowski (Cincinnati Children’s Hospital Medical Centre, OH, USA). AML cell lines were cultured in RPMI medium (Biowest) supplemented with fetal bovine serum (FBS, Lonza), 2 mM L-Glutamine (Lonza) and/or 0.1 mM non-essential amino acids (Lonza) according to manufacturers’ recommendations. Primary MDS and CMML blasts were cultured in IMDM (Biowest) supplemented with 3% heat-inactivated FBS, 2 mM L-Glutamine, 20% BIT 9500 Serum Substitute (StemCell Technologies), 5 ng/ml IL3 (Peprotech), 1 mM sodium pyruvate and 5 × 10^−5^ M β-mercaptoethanol (Sigma-Aldrich,) and 0.1 mM non-essential amino acids (Lonza).

### Drugs and antibodies

All drugs were resuspended in H_2_O or DMSO (Sigma-Aldrich) according to manufacturer’s instructions and were stored at −80 °C at 10 mM (Table S2). Antibody information is found in Table S3.

### Surface phenotype

Ficolled-primary samples were stained for DRD1, DRD2, DRD3, DRD4, DRD5, HTR1A or HTR1B on the surface, using the antibodies listed in Table S3, simultaneously with APC-conjugated anti-human CD45 (clone HI30) (BD Bioscience) and the live-dead discriminator dye 7-AAD (eBioscience). Cells were acquired in a FACSCanto II cytometer (BD). In patient samples, the analysis was performed within the live *blast gate* (CD45^dim^ − SSC^low/int^)^48,49^, whereas HD samples were gated based on CD45^pos^ based on the absent of a blast population, using FlowJo software (TriStar).

### Cytotoxicity assay

4 × 10^4^ cells per mL were cultured in 96-well plates in complete medium and all drugs were added at the indicated concentration. 72 h after, cell viability was measured by 7-AAD (eBioscience) exclusion and Hoechst33342 (Sigma-Aldrich) positivity staining by flow cytometry, and cell count was obtained by volume in a FACSCanto II cytometer (BD). Statistical analysis was calculated in GraphPad (Prism software). FlowJo software (TriStar) was used for flow cytometry analysis.

### Differentiation assay

Primary samples were treated at the indicated drug concentration for 72 h. PE-conjugated anti-human CD11b (clone ICRF44) (BD Pharmingen) was used as differentiation marker. Samples were measured by flow cytometry (FACSCanto II, Becton-Dickinson) and analyzed in FlowJo software (Tristar).

### Clonogenicity assay

50 × 10^3^ primary CMML cells were treated at the indicated concentration for 18 h, and cultured in 1 mL of MethoCult H4034 Optimum (StemCell Technologies). Colonies were screened based on morphology (monocyte/granulocyte-like cells) and cellularity (clusters of >40 cells) at day 14.

### Statistical Analysis

Unpaired two-tailed t student analysis was completed to identify the statistical significance in Figs 2, 4, 5A–D, and 6. Data presented in Figs 1, 2, and 5E–G was analysis using a Mann-Whitney U-test. Statistical analysis was performed using the Prism GraphPad software.

## Electronic supplementary material

Supplementary Material
