# Supplementary material for: Serotonin receptor type 1B constitutes a therapeutic target for MDS and CMML
Source: Sci Rep. 2018 Sep 17;8:13883. doi: 10.1038/s41598-018-32306-4 (PMC6141614; doi:10.1038/s41598-018-32306-4)

Serotonin receptor type 1B constitutes a therapeutic target for MDS and CMML.

Antònia Banús-Mulet[1,2], Amaia Etxabe[1,2], Josep Maria Cornet-Masana[1,3,4], Miguel Ángel Torrente[1,4,5], María Carmen Lara-Castillo[1,6], Laura Palomo[1], Meritxell Nomdedeu[1,5], Marina Díaz-Beyá[1,4,5,7], Francesc Solé[1], Benet Nomdedeu[4,5], Jordi Esteve[1,4,5,7], Ruth M. Risueño[1*]

[1]Josep Carreras Leukaemia Research Institute (IJC). Barcelona, Spain.

[2]Faculty of Pharmacy. University of Barcelona, Spain.

[3]Institut d'Investigació en Ciències de la Salut Germans Trias i Pujol (IGTP). Badalona, Spain.

[4]Faculty of Medicine. University of Barcelona, Spain.

[5]Department of Hematology. Hospital Clínic. Barcelona, Spain.

[6]Leukos Biotech. Barcelona, Spain.

[7]Institut d'Investigacions Biomèdiques August Pi i Sunyer (IDIBAPS). Barcelona, Spain.

RUNNING TITLE: HTR in MDS and CMML

[*] Corresponding author:

Ruth M. Risueño

Josep Carreras Leukaemia Research Institute

Campus Clínic-UB

Carrer del Rosselló, 149-153

08036 – Barcelona (Spain)

Telf: +34 93 227 5400 ext.4528

e-mail: risueno@carrerasresearch.org

SUPPLEMENTARY FIGURES

Table S1. Patients' treatment information. HMA: hypomethylating agents. CT: chemotherapy. Immuno: immunomodulators.

Table S2. Small molecules information.

Table S3. Antibodies information.

Figure S1. DRs' surface expression pattern on MDS cells. DRD1 (**A**), DRD2 (**B**), DRD4 (**C**), and DRD5 (**D**) surface expression measured by flow cytometry in blood samples from healthy donors (HD), MDS samples and AML samples. Results are graphed as a box plot, the statistical median is indicated as a horizontal line, error bars correspond to SEM. HD n=4-6; MDS n=71-90; AML n=14. ** p<0.01; *** p<0.001; **** p<0.0001.

Figure S2. Symbol legend of MDS samples used in the experiments. Each symbol type corresponds to an MDS patient sample. Healthy donors (HD), grey; RS-MLD, light purple; RS-SLD, dark purple; 5q-, red; MLD, orange; EB-1, light blue; EB-2, dark blue.

Figure S3. Expression of DRs and HTRs in CD45^{low} and CD34^{+} MDS cell subpopulations. (**A**) HTR1A, HTR1B, DRD3 and DRD5 surface expression in CD45^{low} and CD34^{+} subpopulations in MDS samples (n=23). (**B**) HTR1A and HTR1B surface expression within the CD34^{+} cell population in healthy donor (HD) and MDS samples (MDS). Horizonal lines represent grand means. HD n= 3; MDS n= 19. *** p<0.001.

Figure S4. DR's and HTR's antagonists induce cell death and differentiation on MDS cells. MDS patient samples were treated with 10 μM apomorphine (apo) and 10 μM SB224289 (SB9) 72 h. (**A**) Frequency of CD11b-positive cells measured by flow in vehicle control-, apomorphine- and SB9-treated MDS samples. N=2 in triplicates. (**B**) Representative flow plot of CD11b surface staining. MDS patient samples were treated with 50 μM thioridazine (thio). (**C**) Cell viability was measured by 7-AAD exclusion by flow cytometry, (**D**) differentiation status as determined by CD11b expression by flow cytometry, and (**E**) a representative flow plot

of each condition is presented. Each symbol type corresponds to a patient sample, and each symbol corresponds to an experimental point. * $p<0.1$, ** $p<0.001$; **** $p<0.0001$.

Figure S5. MDS-L do not express DRs or HTR1s. HTR1A (**A**), HTR1B (**B**), DRD3 (**C**) and DRD5 (**D**) surface expression levels in MDS-L (solid line) compared to unstained negative control (tinted line) measured by flow cytometry. A representative histogram is shown.

Figure S6. HTR and DR antagonists synergize with azacytidine and decitabine. Synergistic effect of the co-treatment with (apomorphine (apo) and methiothepin (methio) with (**A**)(**C**) azacytidine (aza) or (**B**)(**D**) decitabine (deci) as measured by (**A**)(**B**) EOBA and (**C**)(**D**) CI in HL-60 AML cells.

Sample code	HMA/CT/Immuno	Other treatment
MDS #1	No	No
MDS #5	No	No
MDS #6	No	No
MDS #9	No	No
MDS #10	Yes	No
MDS #12	No	No
MDS #14	No	No
MDS #15	No	No
MDS #16	No	No
MDS #17	No	No
MDS #20	No	No
MDS #23	No	No
MDS #24	Yes	No
MDS #26	No	No
MDS #27	No	No
MDS #28	No	No
MDS #29	No	No
MDS #30	No	No
MDS #31	Yes	No
MDS #32	No	No
MDS #33	Yes	No
MDS #34	Yes	No
MDS #35	No	No
MDS #36	No	No
MDS #37	No	No
MDS #38	No	No
MDS #39	No	No
MDS #40	Yes	No
MDS #41	No	No
MDS #43	No	Yes
MDS #49	Yes	No
MDS #50	Yes	No
MDS #52	Yes	No
MDS #53	No	Yes
MDS #57	No	No
MDS #60	Yes	No
MDS #68	No	No
MDS#73	No	No
MDS #74	No	No
MDS #76	Yes	No

MDS #78	No	Yes
MDS#80	Yes	No
MDS #93	No	No
MDS#95	Yes	Yes
MDS #98	Yes	No
MDS#103	N/A	N/A
MDS#110	N/A	N/A
MDS#113	N/A	Yes
MDS #119	Yes	No
MDS#122	No	Yes
MDS #131	No	No
MDS #132	No	No
MDS #134	No	No
MDS #138	Yes	No
MDS #140	No	No
MDS #143	No	Yes
MDS #145	Yes	No
MDS #149	Yes	No
MDS #151	Yes	No
MDS #154	No	No
MDS#161	N/A	No
MDS #162	Yes	No
MDS #166	Yes	No
MDS #170	Yes	No
MDS #176	No	Yes
MDS #181	No	Yes
MDS #186	No	No
MDS #187	No	Yes
MDS #188	No	No
MDS #195	Yes	No
MDS#197	No	No
MDS #210	No	No
MDS #211	No	No
MDS #212	No	No
MDS #213	No	No
MDS #214	No	No

Name	Abbreviations	Comercialized by	Target	Reference
Apomorphine	Apo	Sigma-aldrich	HTR1/2 antagonist	Millan, 2002
Methiothepin mesylate	Methio	Santa-Cruz Biotechnology	HTR1/2 antagonist	Monachon, 1972
NAN-190 hydrobromide	NAN190	Sigma-aldrich	HTR1A antagonist	Glennon, 1988
SB224289	SB9	Biogen	HTR1B antagonist	Selkirk, 1998
SCH-23390	SC90	Biogen	D1/D5 antagonist	Millan, 2001
UH-232	UH232	Biogen	D2 antagonist	Svensson, 1986
Thioridazine	Thio	Sigma-aldrich	PanDR antagonist	Sunahara, 1991
Chlorpromazine hydrochloride	CPZ	Sigma-aldrich	PanDR antagonist	Freedman, 1994
Decitabine	Deci	Sigma-aldrich	Hypomethylating agents	Jones, 1980
Azacitidine	Aza	Sigma-aldrich	Hypomethylating agents	Sorm, 1964

Antibody	Reference	Comercialized by	Conjugated fluorochrome
Anti-5HT1A receptor	AB 85615	ABCAM	-
Anti-5HT1B receptor	ABIN738021	Antibodies-online	-
Anti-DRD1 receptor	324390	EMD Millipore	-
Anti-DRD2 receptor	324393	EMD Millipore	-
Anti-DRD3 receptor	324402	EMD Millipore	
Anti-DRD4 receptor	324405	EMD Millipore	-
Anti-DRD5 receptor	324408	EMD Millipore	-
CD11b/Mac-1	ICRF44	BD	PE
CD45	HI30	BD	FITC, PE, APC, V450

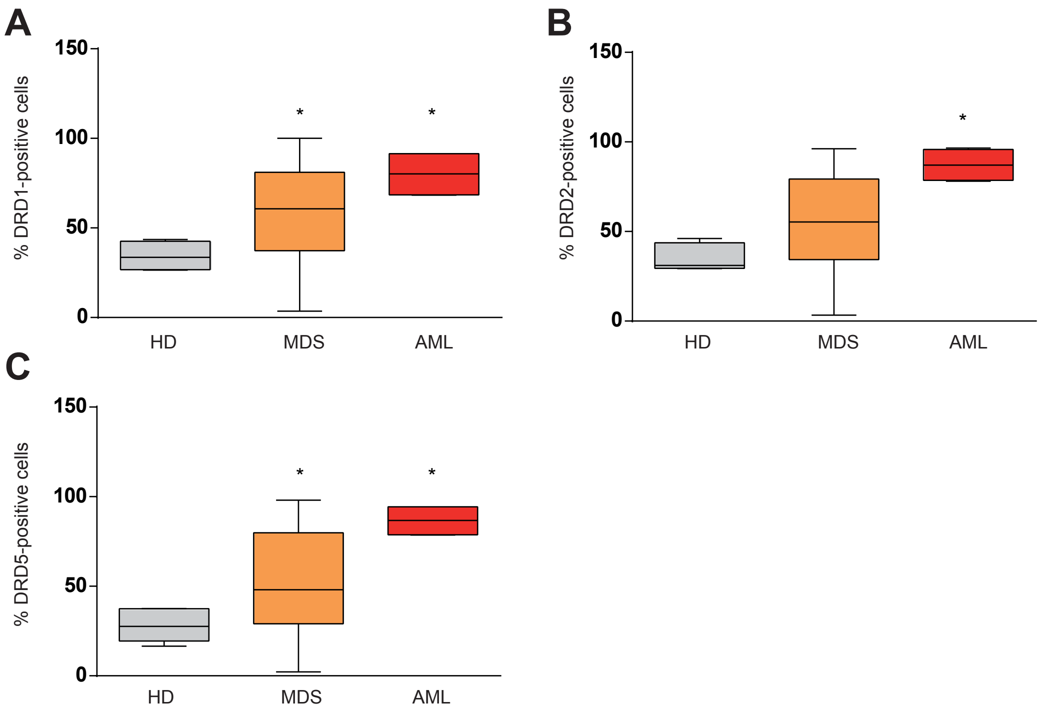
A
150
100
50
0
% DRD1-positive cells
*
*
HD
MDS
AML
B
150
100
50
0
% DRD2-positive cells
*
HD
MDS
AML
C
150
100
50
0
% DRD5-positive cells
*
*
HD
MDS
AML

Banús-Mulet *et al*. Supplementary Figure 2

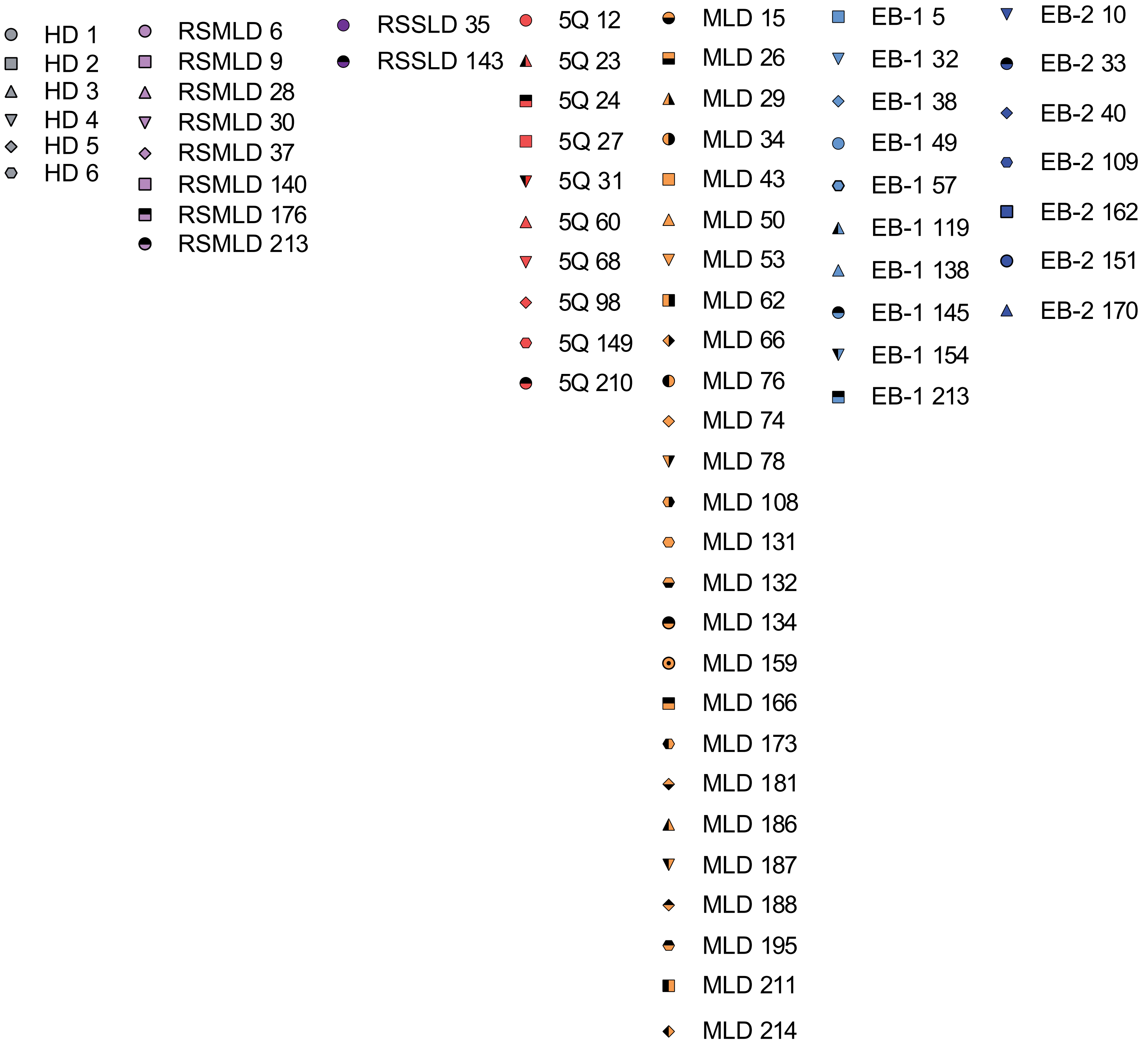

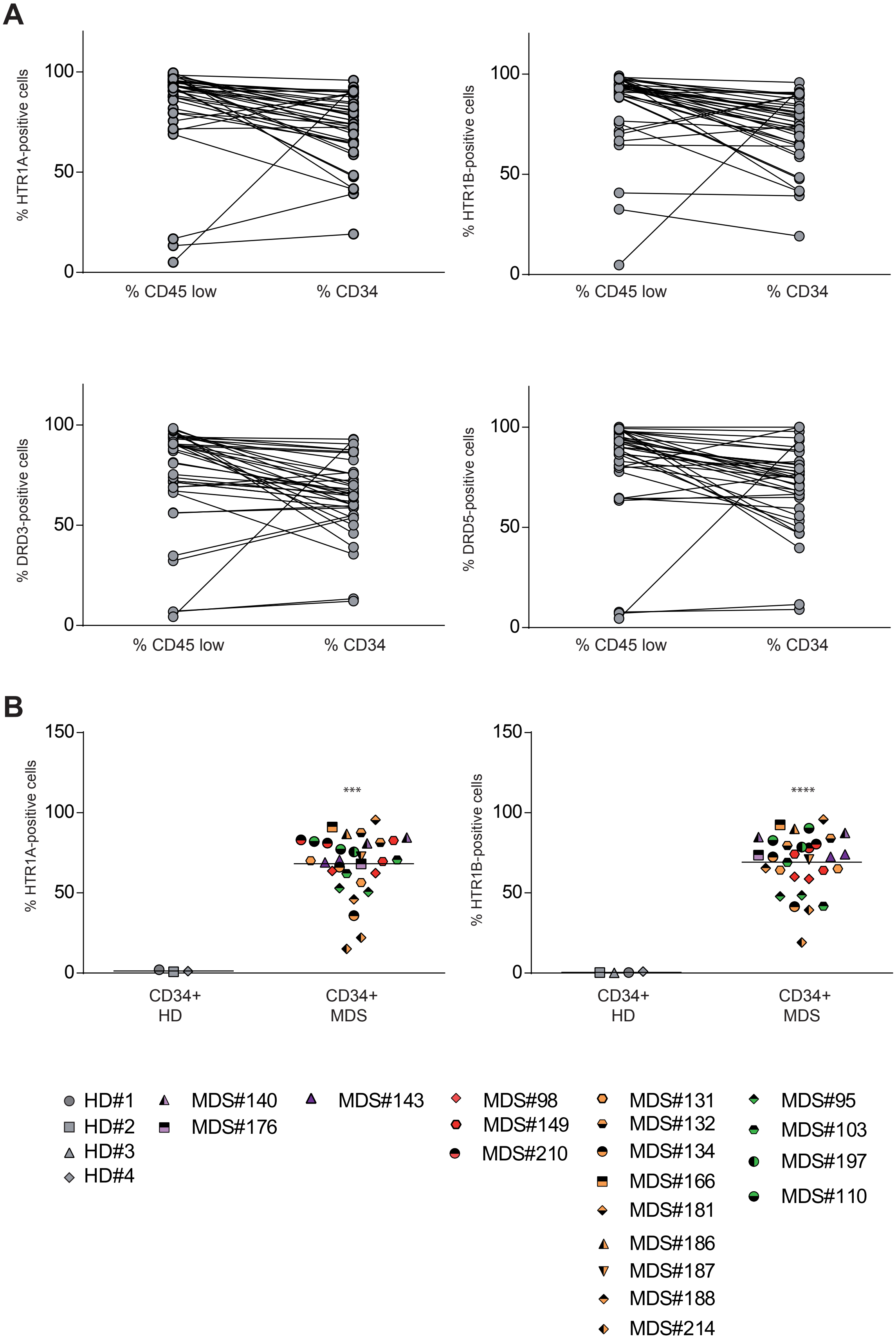

A
% HTR1A-positive cells
100
50
0
% CD45 low
% CD34
% HTR1B-positive cells
% DRD3-positive cells
% DRD5-positive cells
B
150
***
****
% HTR1A-positive cells
% HTR1B-positive cells
CD34+
HD
CD34+
MDS
HD#1
HD#2
HD#3
HD#4
MDS#140
MDS#176
MDS#143
MDS#98
MDS#149
MDS#210
MDS#131
MDS#132
MDS#134
MDS#166
MDS#181
MDS#186
MDS#187
MDS#188
MDS#214
MDS#95
MDS#103
MDS#197
MDS#110

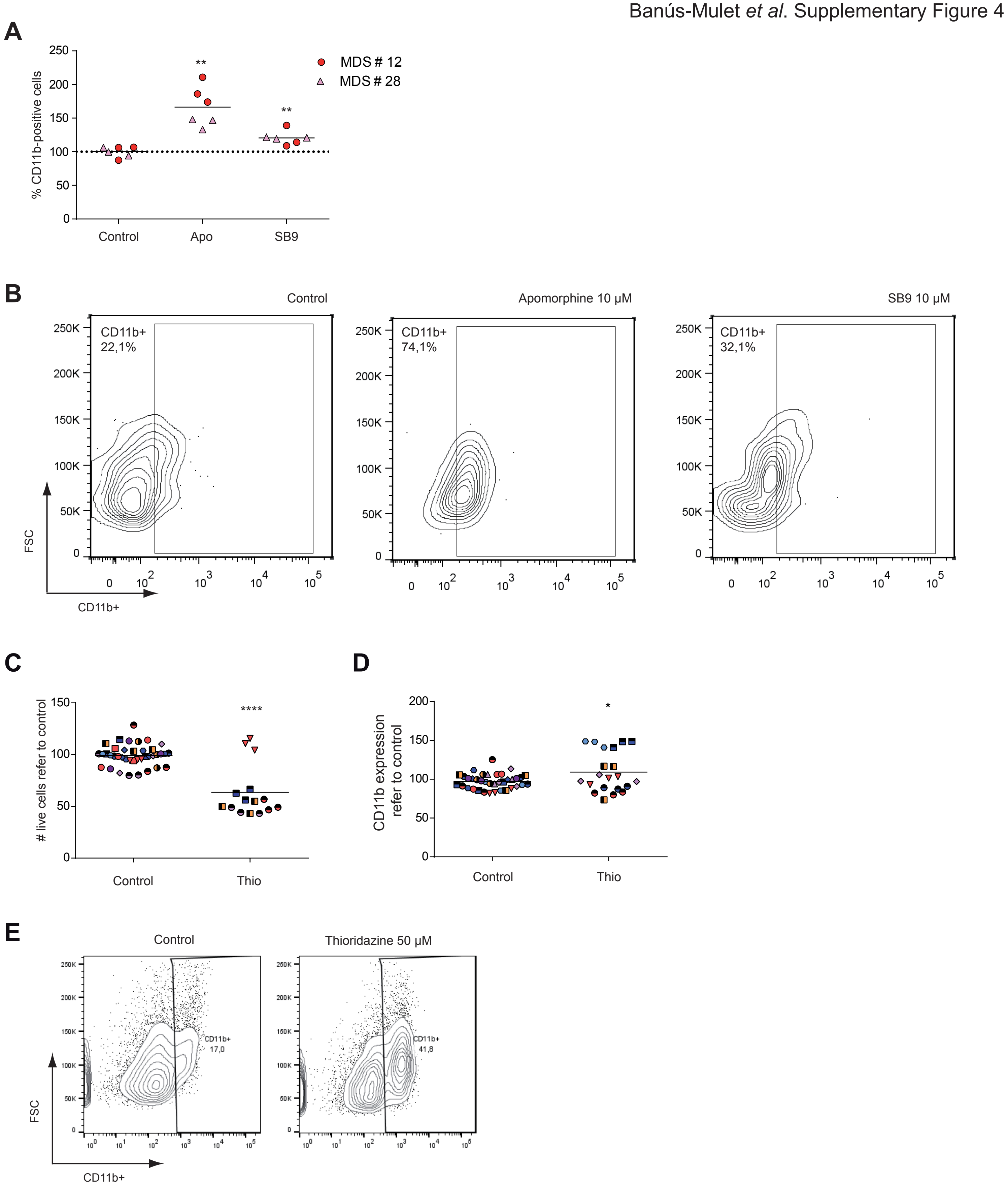
A
% CD11b-positive cells
250
200
150
100
50
0
Control
Apo
SB9
**
**
MDS # 12
MDS # 28
B
Control
Apomorphine 10 µM
SB9 10 µM
CD11b+
22,1%
CD11b+
74,1%
CD11b+
32,1%
250K
200K
150K
100K
50K
0
FSC
CD11b+
C
live cells refer to control
150
100
50
0
Control
Thio
****
D
CD11b expression
refer to control
200
150
100
50
0
Control
Thio
*
E
Control
Thioridazine 50 µM
CD11b+
17,0
CD11b+
41,8
FSC
CD11b+

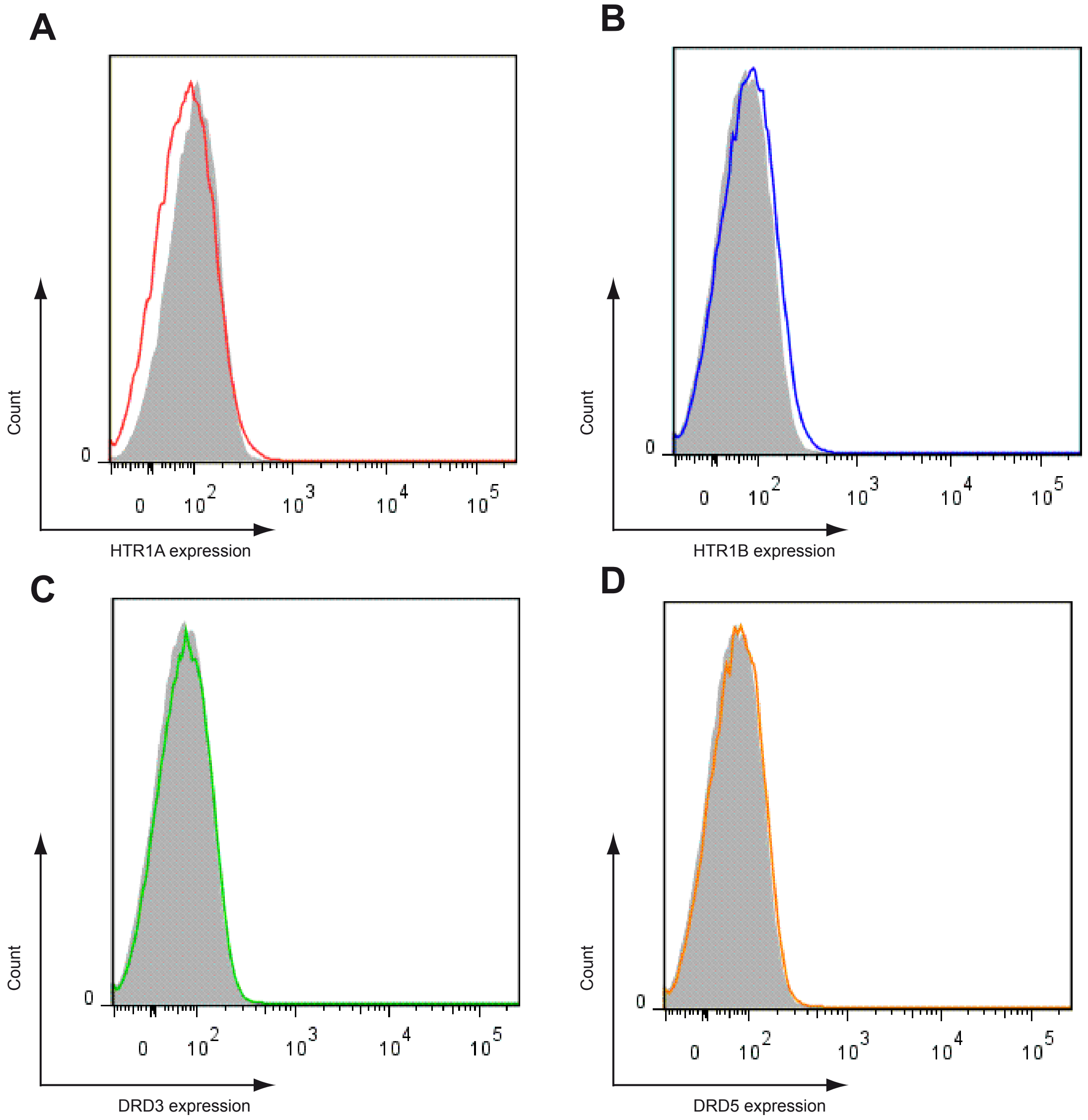
A
B
C
D
Count
0
0
10^2
10^3
10^4
10^5
HTR1A expression
HTR1B expression
DRD3 expression
DRD5 expression

Banús-Mulet *et al*. Supplementary Figure 6

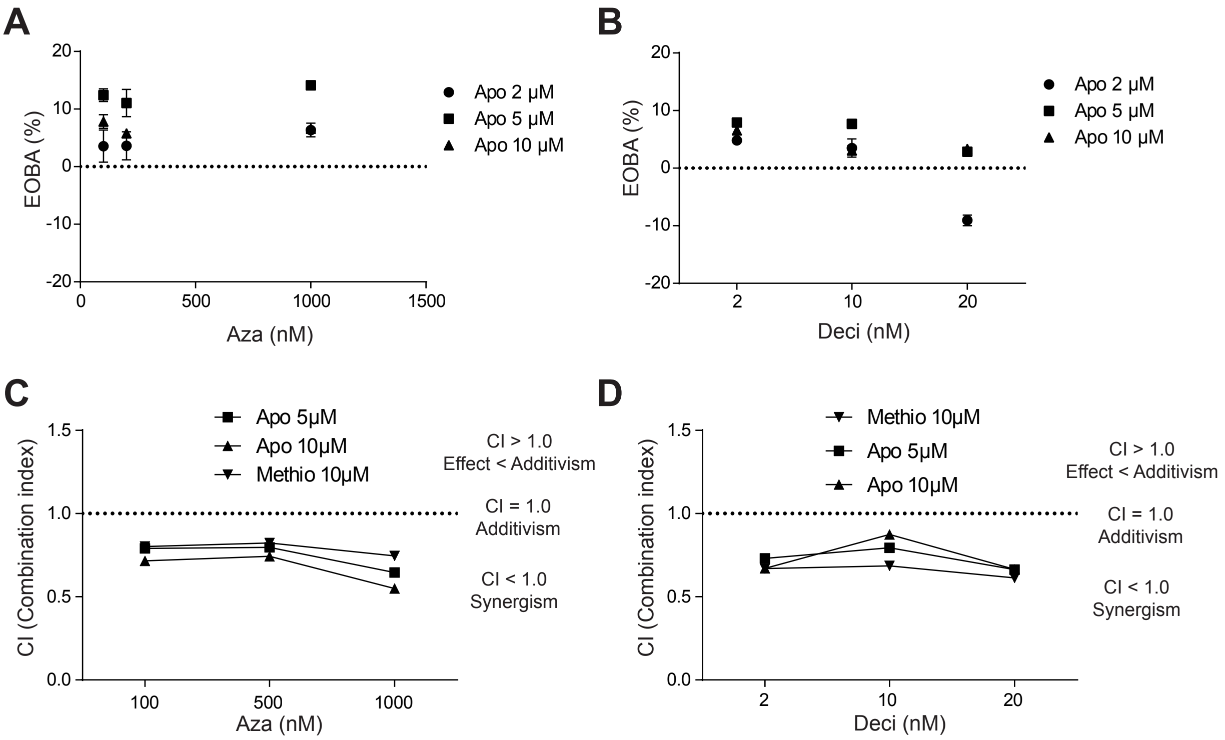

Supplement: Supplementary file 1 — Supplementary Material [file 41598_2018_32306_MOESM1_ESM.pdf]
